# Relative income concerns and smoking behaviour: The role of unobserved heterogeneity

**DOI:** 10.1371/journal.pone.0295333

**Published:** 2024-03-14

**Authors:** Alpaslan Akay, Asena Caner

**Affiliations:** 1 Department of Economics, University of Gothenburg, Gothenburg, Sweden; 2 Department of Economics, Bahçeşehir University, Istanbul, Türkiye; 3 Department of Economics, TOBB University of Economics and Technology, Ankara, Türkiye; University of Jyvaskyla, FINLAND

## Abstract

Status or relative concerns (as in the idiom ‘keeping up with the Joneses’) can lead to negative feelings such as stress and anxiety. One key question is whether these concerns relate to daily smoking behaviour. The conjecture is that status concerns and the accompanying stress and anxiety might be associated with a higher likelihood of smoking and a higher number of cigarettes smoked, generating a higher instant physical reward and reducing the stress and anxiety. The literature aiming to identify this relationship focuses mostly on a single cross section of individuals, ignoring potential differences in unobserved characteristics of smokers and non-smokers (e.g., genetic factors, personality differences, parental smoking during childhood). This paper investigates the role of unobserved individual characteristics on this relationship, which has not been done in previous studies. Using a long panel data of smoking information in Germany and a variety of panel data model specifications, we show that there is no statistically significant association between relative income concerns and the likelihood of smoking or the number of cigarettes smoked among the overall population. We find a positive and significant relationship only among people who smoked at least one cigarette in the past. A 10% appreciation in the income of comparable other individuals relates to about 3.5 more cigarettes per month among these people. Importantly, failing to allow for the unobserved influences of smoking leads to three times larger estimates than when using models with unobserved factors correlating to the income and smoking behaviour. The results are robust with respect to alternative assumptions and specifications where we use different functional forms of unobserved heterogeneity, definitions of relative concerns, incomes, and reference groups.

## Introduction

Smoking tobacco products (e.g., cigarettes, pipes, cigars) is beyond doubt bad for health. Yet, people take the health risk of smoking even though the harmful effects on health are well known. One key area of research aims to understand the determinants of such risky behaviours (e.g., [1–3]). A hidden correlate tied to an important source of stress and anxiety might be income comparisons or status concerns, which lower life satisfaction or happiness and may induce mental health problems (e.g., [4, 5]). The conjecture is that as status concerns raise stress or anxiety, a person might be tempted to seek relief and pleasure from the instantaneous reward of nicotine and smoke more or relapse after quitting. It is reported in the medical literature that nicotine can modulate the function of pathways in the brain involved in stress response, anxiety, and depression ([6, 7]). The literature investigating the income comparisons and smoking behaviour is mostly limited to cross-sectional data that ignores unobserved individual factors (e.g., [8, 9]). Yet, failing to allow for unobserved individual factors might induce bias on the estimated relationship between relative income and smoking if these factors are correlated with the income sources and smoking behaviour. Indeed, the literature identifies important unobserved differences between those who become addicted to nicotine and others. These differences might be deeply related to genetic predisposition, personality characteristics, beliefs, or situational influences ([10–12]). Biological differences between individuals in sensitivity or response to nicotine may account for some of the genetically influenced inclination to become an addicted smoker. Some dimensions of personality, risk-taking, and other reward- and sensation-seeking behaviours tend to reduce sensitivity to potential damage and promote cognitive, social, and behavioural actions that reinforce smoking. Findings indicate that the genetic disposition to smoke is influenced in part by individual differences in personality and psychopathology. According to Gilbert and Gilbert ([11]), “As much as half of the genetics of smoking may be explained by the major personality factors (neuroticism, extraversion, psychoticism, agreeableness, and conscientiousness); a substantial additional portion is also very likely mediated by intelligence and socioeconomic status” (p. 144, [11]).

To the best of our knowledge, this study is among the first to use a long panel of smoking information and panel data fixed-effects model specifications to investigate how relative concerns relate to smoking behaviour. A small number of studies have turned to panel data analysis to account for individual-level unobserved effects to investigate smoking behaviour. Using the Socio-Economic Panel data, Heilert and Kaul ([13]) examine how smoking prevalence and cigarette consumption changed over time (during 1998–2014) in socio-economic subgroups defined by gender, age, education, and income; Monfared and others ([14]) study the longitudinal changes in smoking behaviour and their association with personal factors (such as self-control, mental health, and socio-economic status) versus the behaviour of other household members. Other papers use panel data to study smoking behaviour in specific age groups. Using data from the 2003–2007 Korea Youth Panel Study, Do and Finkelstein ([15]) test whether higher employment income or allowance leads youth to smoke. Lahiri and Li ([16]) use longitudinal data from Health and Retirement Surveys over 1992–2010 to analyse how cigarette prices, health shocks, and smoke-free laws affect smoking decisions by older Americans.

We show that failing to allow for individual unobserved differences between individuals leads to biased estimates and wrong policy conclusions. The empirical strategy in this paper is based on the Socio-Economic Panel (SOEP), a long panel dataset that covers individuals’ cigarette smoking behaviour biennially from 2002 to 2018 (nine waves). Our econometric approach follows the conceptual framework adopted by the extant empirical literature on the determinants of smoking, which has shown a close association between socio-economic status and smoking, regardless of how these variables are defined (see, for instance, [17–20]). We trace individuals through the years and identify the group of individuals with whom they compare their income (i.e., reference group). The methodology of generating reference groups adopted in this paper follows the empirical literature (in particular, subjective well-being literature) that investigates relative concerns (e.g., [21–23]). Recent empirical subjective well-being and experimental literature reports that income comparisons form negative externalities that lead to welfare losses (e.g., [21, 24]) as well as important implications on immigration ([25]), health policy ([26]), poverty ([27]), economic growth ([28]), optimal taxation ([29]), labour supply (e.g., [30]), and sleep behaviour ([23]).

The empirical strategy to identify the time-variant unobserved differences is based on the fixed-effects model specifications, where we capture the effects of absolute and relative income on the probability and the number of cigarettes smoked among the whole population and among people who smoked at least once during the sampling period. To obtain comparable tobacco consumption measures, we focus only on cigarette consumption and exclude other forms of tobacco use (e.g., cigars and pipes). To investigate the role of time-invariant unobserved heterogeneity, we present results from pooled regressions in comparison with the fixed-effects (and alternative) specifications. Our cross-sectional estimates are similar to the findings reported in the literature (cf. [9]), suggesting that there is a positive and strong association between relative concerns and smoking. Yet, our fixed-effects specifications suggest that allowing for the unobserved individual effects (capturing time-invariant measures such as genetic differences) completely eliminates the cross-sectional relationship among the whole population. We find a significant relationship only among people who smoked cigarettes at least once in their life reflecting the importance of the unobserved differences between smokers and non- smokers. The magnitude of the relationship among people who have smoked at least once is 3.5 cigarettes/month for a 10% appreciation in the income of comparable other individuals. The magnitude is highly overestimated with models failing to allow for unobserved heterogeneity (up to three times larger estimates).

We also investigate the relative concerns among people who are relatively deprived (poorer people who make upward comparisons) and people who are relatively better off (richer people who make downward comparisons). As in most studies in the literature, failure to allow for the unobserved individual characteristics leads a substantial overestimation of the relationship and wrong conclusions about which group of people was actually affected by the relative concerns (cf. [9]). The fixed-effects model specification suggests that the relationship is statistically significant only among the poor (upward comparators or relatively deprived), as expected. The results are found to be highly robust with respect to estimators, treatment of unobserved individual effects, accounting for time-invariant personality differences, life satisfaction, risk-taking behaviour, income sources, income inequality, and alternative reference groups.

The remainder of the paper is organised as follows. The next section describes the empirical approach and the data, gives descriptive statistics, and presents the econometric model. In our results section, we present the main results and an extensive robustness analyses. In the final section, we conclude the paper.

## Empirical approach

### The data and selection

The panel data at use are from the Socio-Economic Panel (SOEP, for further information about the data, see www.diw.de and https://paneldata.org/). The dataset includes large and representative panels of households interviewed since 1984 and has low attrition rate ([31]). Each wave includes about 12,000–15,000 households from both East and West Germany. Our sample selection criterion is straightforward; we include all individuals of ages 18–80 in all waves where information on smoking is available. Following the literature on the determinants of smoking behaviour, we employ a large selection of control variables in our model specifications, including socio-demographic and economic characteristics of individuals, household composition, and health and risk measures, among many others.

### Measures and descriptive statistics

#### Smoking behaviour

The first set of key measures used in this study is on *cigarette smoking* behaviour. The dataset includes information on cigarette smoking behaviour, biennially from 1998 to 2018. We use two measures of smoking: first, whether the individual smokes cigarettes or not, and second, the number of cigarettes smoked per day. The questionnaires in waves 1998 and 2000 asked for the number of cigarettes, cigars, and pipes smoked altogether; therefore, the consumption amounts of different tobacco products are unidentifiable. In 2002 to 2018, cigarette smoking was asked separately from the consumption of other tobacco products. In this paper, we focus only on cigarette consumption. The reasons for this choice are as follows: First, cigarette smoking is the predominant method of tobacco consumption all over the world. Indeed, our sample also suggests that while 28.2% of people in the sample smoke cigarettes, 0.4% regularly smokes pipe and 0.9% smoke cigars. In line with Heilert and Kaul ([13]), 95.6% of all smokers in Germany smoke cigarettes exclusively. Second, it is easier to identify the amount of tobacco consumption for cigarettes than for cigars and pipes. The amount of tobacco used in a cigar or per pipe might show more variation than the amount of tobacco used in a cigarette on average across brands in the market or those that are self-rolled.

The final sample used in the estimations includes 179,312 individual × year observations consisting of 50,512 individual × year observations reporting as ‘currently smoking’. We use this information to define our first dependent variable, which is a dummy indicating those who are currently smoking. The second dependent variable is the number of cigarettes smoked. This dependent variable takes a value of 0 if an individual is not smoking at all or a positive value if an individual smokes one or more cigarettes per day. There are some extreme observations in the dataset. We drop the 14 observations for which the number of cigarettes smoked is greater than 90. We also consider a third dependent variable by identifying a sample of 63,490 individual × year observations consisting of individuals who smoked at least one cigarette in one of the waves from 2002 to 2018. Among them, there are 14,984 individual × year observations with zero cigarette consumption, who were smokers but did not smoke in the survey year (i.e., past smokers or quitters), or people who relapsed (i.e., people who quit smoking but started to smoke again). The biennial dataset allows us to observe cigarette smoking behaviour of individuals every two years. Thus, the biennial nature of the data reduces the sample size. However, with a quite large sample, this does not create a big concern. We note that all other characteristics of individuals are available annually. We will allow income lags to capture the relationship between how income experienced in previous period relate to the period when cigarette smoking behaviour is observed. Detailed results will be presented in our robustness analysis.

#### Income, reference groups, and comparison measures

The baseline income measure used in this study is the total (post-governmental) household income. The *absolute income* measure is the total net income enjoyed by the household members after all tax payments and governmental benefits (including all social security transfers). Our ‘relative income’ measure is defined using the total household income. To this end, we calculate the mean income of ‘comparable other individuals’ as a measure for the ‘reference income’ or ‘comparison income’ point. The definition of a reference group involves rather abstract and dynamic psychological, social, and cultural aspects ([32]). The reference groups might also be multiple, implying that individuals make comparisons with different groups of people simultaneously, which can also differ by specific goods. For instance, salaried workers might make comparisons with other salaried workers as well as self-employed individuals. As Falk and Knell ([33]) discuss, individuals might also choose their reference groups consistent with their life goals and well-being, generating endogeneity issues. The reference group, with whom the individuals compare their income, is defined by using some criteria. Following the bulk of the literature, we select spatial criteria including the period and region of observation as well as individuals’ socio-economic characteristics (e.g., [21–23]). The baseline reference groups are defined using the following criteria: “*all individuals who are living in the same region (West or East Germany), with the same age ([20, 30], (30,40], (40,50] and (50,65]), the same gender (male or female), and the same skill levels (low and high) every period of observation*”. We split our sample using the International Standard Classification of Education (ISCED) score. There are six skill classifications in ISCED given as follows: 1) no or inadequate schooling/in school; 2) general elementary; 3) middle vocational; 4) vocational plus abitur; 5) higher vocational; and 6) higher education. We group the first three categories as low-skill (i.e., low level of education) and the rest as high-skill (i.e., high level of education). The number of reference groups with these criteria is 432, and the mean number of individuals is 730.8, with standard deviation (s.d.) 385.3.

The *relative income* measure is then captured by using the mean household income calculated within each reference group. The measure is time-variant, and the relationship between relative income and smoking can be identified within fixed-effects framework. To check the robustness, we generate another relative income measure using *median* household income within the baseline reference groups. We also use alternative measures of relative concerns that are based on *ranks* within the reference group, as well as alternative criteria in the reference groups (e.g., marital status and finer spatial units). As a robustness check, we experiment with equivalised income, considering the household size and how the income is brought to the household. We calculate the equivalised per capita household income enjoyed by each individual using the standard OECD scales (the model’s specifications also allow for a rich set of variables for the household composition). Several other checks are presented in our robustness analysis, covering windfall income, previous year income (lags) and income inequality within reference groups (i.e., within reference groups’ Gini measures), and reference group definitions (i.e., alternative criteria).

#### Descriptive statistics

Table 1 presents descriptive statistics of key control variables used in our empirical analysis. In the full sample, the mean number of cigarettes smoked per day is about 4.1 (s.d. 8.13). Among ‘currently smoking’ individuals in wave *t*, the mean number of cigarettes smoked is about 14.6 cigarettes/day (s.d. 9.1). The sample that uses individuals who smoked at least one cigarette during the sampling period (2002–2018) captures all time periods of these individuals whether they smoked in a particular wave *t* or not. This sample includes people who smoked during all waves, as well as those who quit at some point, or resumed smoking (i.e., relapsed). The mean number of cigarettes smoked in this sample is 11.6 (s.d. 10.0). The absolute income measure (post-governmental net household income in 10,000 euros) suggests that smokers have a lower level of income compared with non-smokers (4.26 vs. 3.69). Yet, the share of currently employed individuals is about 10 percentage points higher among people who are currently smoking or who smoked at least once (0.64 vs. 0.75). They also work about five hours more during an average week (22.8 vs. 26.3). Smokers are predominantly younger and single males. They are also slightly less educated and the share of home ownership is lower. In our regressions, we use several other control variables, which include the usual socio-demographic and socio-economic individual and household characteristics: marital status (married/cohabiting, single, divorced, or widowed), number of children aged 0–1, 2–4, 5–7, 8–10, 10–12, 13–15, and 16–18, health status (five categories), disability status, capital income or non-labour income (rent, dividend, and interest income), and region of residence (16 federal states).

**Table 1:** Descriptive statistics.

	Whole Sample	Currently Non-Smoker	Currently Smoker	Smoked at least one cigarette during 2002–2018
#Cigarettes smoked	4.105		14.572	11.593
	(8.131)		(9.064)	(9.995)
Absolute income (yearly/10,000 Euro)	4.098	4.259	3.687	3.764
	(3.212)	(3.255)	(3.063)	(3.025)
Employment status (active = 1)	0.672	0.643	0.746	0.744
	(0.469)	(0.479)	(0.435)	(0.436)
Working hours	23.061	21.78	26.325	26.142
	(20.676)	(20.534)	(20.678)	(20.615)
Age in years	49.035	50.719	44.742	45.034
	(15.566)	(15.904)	(13.766)	(13.882)
Gender (female = 1)	0.525	0.549	0.464	0.479
	(0.499)	(0.498)	(0.499)	(0.500)
Marital status (married = 1)	0.637	0.677	0.536	0.552
	(0.481)	(0.468)	(0.499)	(0.497)
Marital status (single = 1)	0.205	0.182	0.265	0.256
	(0.404)	(0.386)	(0.441)	(0.436)
Years of education	12.347	12.596	11.712	11.821
	(2.713)	(2.795)	(2.374)	(2.427)
Living in West Germany (West = 1)	0.771	0.772	0.766	0.768
	(0.420)	(0.419)	(0.423)	(0.422)
Household size	2.757	2.748	2.78	2.773
	(1.309)	(1.291)	(1.354)	(1.333)
House ownership (owner = 1)	0.524	0.576	0.39	0.408
	(0.499)	(0.494)	(0.488)	(0.491)
#Observations	179,312	128,797	50,512	63,490

Authors’ own calculations from the SOEP data (2002–2018). Standard deviations are presented in parentheses.

### Econometric specifications

#### The model

In our model specifications, the dependent variable *S*_*it*_ for individual *i* observed in wave *t* takes two forms: it is either a dummy of being a smoker or the number of cigarettes smoked. The generic model specification is

$$\begin{array}{c} S_{it}=\phi_{R_{j}}ln\left(Y_{i,t}^{R_{j}}\right)+\phi_{A}ln\left(Y_{i,t}\right)+X^{\prime }\gamma +\epsilon_{it}, \end{array}$$ (1)

$$\begin{array}{c} \epsilon_{it}=W_{t}+\rho_{r}+\alpha_{i}+\varepsilon_{it}, \end{array}$$ (2)

where the key variables of interest are the absolute income *Y*_*it*_ and relative income $Y_{it}^{R_{j}}$ of the individual. To allow for the diminishing returns of absolute and relative income, we use logarithmic transformation for the income measures. The relative income is the *mean income* in the reference group *R*_*j*_,—that is, the ‘reference or comparison income point’ of individual *i* at year *t* as $Y_{it}^{R_{j}}=\frac{1}{N_{R_{j}}-1}\sum_{i=1}^{N_{R_{j}}-1}Y_{it}$, where $N_{R_{j}}$ is the number of individuals within the reference group *R*_*j*_, which is determined by the criteria used to define the reference group (e.g., region, age, gender, and skill levels every year). We are mainly interested in the parameter $\phi_{R_{j}}$ which shows how mean income of comparable other individuals relates to the probability of smoking or the number of cigarette smoked per day. The expected sign of $\phi_{R_{j}}$ is positive according to our main conjecture stating that a higher level of relative income positively relates to the smoking behaviour.

#### The asymmetrical specification

We also allow for asymmetry in the relative income $\phi_{R_{j}}$ parameter for those who are making *upward* and *downward* comparisons. To this end, we generate a dummy variable taking the value of one (*Z*_*it*_ = 1) if the individuals’ income is less than the income level of individuals’ reference income ($Y_{it}<Y_{it}^{R_{j}}$) and zero for individuals whose income is higher than their reference income ($Y_{it}\geq Y_{it}^{R_{j}}$). We specify an interaction model by implementing $\psi Z_{it}+\phi_{R_{j}}^{Z=1}Z_{it}ln\left(Y_{it}^{R_{j}}\right)+\phi_{R_{j}}^{Z=0}\left(1-Z_{it}\right)ln\left(Y_{it}^{R_{j}}\right)$ in Eq (1). This allows us to test whether the relative income makes a difference regarding smoking for those who are relatively deprived versus those who are better off compared to the income of others ($H_{0}:\phi_{R_{j}}^{Z=0}=\phi_{R_{j}}^{Z=1}$).

#### Model specifications and estimators

We use a rich set of control variables in our model specifications, which are given in matrix *X*, and *γ* is the corresponding vector of parameters to be estimated. *ϵ*_*it*_ is the error term involving several error components as given in Eq (2). The composite error terms in Eq (2) include wave-specific dummies *W*_*t*_ to capture economy-wide shocks in Germany. To capture unobserved differences across regions, the specification includes region dummies *ρ*_*r*_ for the 16 German federal states (*Länder*). To be able to allow for unobserved characteristics that are correlated with the observed characteristics, we allow for the individual fixed effects *α*_*i*_. These variables are considered to be time-invariant and aim to capture individual specific unobserved confounders (e.g., genetic predisposition and personality). The model specification is estimated via a linear probability model for the probability of smoking and a linear fixed-effects model for the number of cigarettes smoked. We then explore the nature of the dependent variables and estimate non-linear models (e.g., fixed-effects logit for probability and Poisson fixed-effects model for count data) to check the sensitivity of the main results.

## Results

### Main results

#### Probability of smoking

The main results are given in Table 2. We mainly focus on the relative income parameter estimates (the table also briefly presents the parameter estimates of absolute income). We present estimates from both the cross-sectional (upper part of the table) and panel data fixed-effects linear probability (lower part of the table) models. Columns A(I) and A(2) present the estimates from linear probability models where the dependent variable is a dummy indicating individuals who are current smokers at wave *t*. The cross-sectional model (pooled regressions) suggests that the relative income coefficient (Column A(I)) is positive and highly statistically significant (0.144, s.e. 0.018), whereas the estimated relative income coefficient on the probability of smoking is very small in magnitude and statistically imprecise with the fixed-effects model (–0.002, s.e. 0.007). That is, allowing for the unobserved characteristics of individuals that are correlated with the observed characteristics completely invalidates the results from the cross-sectional model.

**Table 2:** Main results: Smoking behaviour and relative concerns.

Dependent Variable:	A(I)	A(II)	B(I)	B(II)	C(I)	C(II)
	Probability of Smoking (Whole Sample)		#Cigarettes (Whole Sample)		#Cigarettes (Smoked at least once during 2002-2018)	
	Baseline	Asymmetric Specification	Baseline	Asymmetric Specification	Baseline	Asymmetric Specification
*Cross-Sectional Specification*						
Absolute income	-0.0039	-0.0081 **	-0.0105	-0.0717	-0.1359	-0.0997
	(0.0034)	(0.0039)	(0.0605)	(0.0720)	(0.1161)	(0.1289)
Relative income	0.1439 ***		2.7766 ***		3.5695 ***	
	(0.0178)		(0.3460)		(0.4474)	
a. Relative income (Poorer)		0.1509 ***		2.8939 ***		3.6299 ***
		(0.0191)		(0.3696)		(0.4436)
b. Relative income (Richer)		0.1349 ***		2.5933 ***		3.1538 ***
		(0.0153)		(0.2865)		(0.5994)
*P-value of the difference (a-b)*		0.1923		0.1505		0.2707
Estimated Magnitudes	5.11		8.33		11.71	
*Panel Data with Individual Fixed Effects*						
Absolute income	-0.0003	-0.0997	0.0589	0.0471	0.1607 **	0.1403 *
	(0.0022)	(0.1289)	(0.0329)	(0.0361)	(0.0801)	(0.0851)
Relative income	-0.0015		0.2422		1.1669 ***	
	(0.0071)		(0.1499)		(0.3631)	
a. Relative income (Poorer)		-0.0044		0.2719 *		1.3994 ***
		(0.0075)		(0.1563)		(0.0801)
b. Relative income (Richer)		0.0019		0.2156		0.4776
		(0.0082)		(0.1662)		(0.4656)
*P-value of the difference (a-b)*		0.3112		0.6066		0.0099
Estimated Magnitudes	-0.05		0.32		3.50	
#Observations	179,312	179,312	179,312	179,312	63,490	63,490

Authors’ own calculations from the SOEP data (2002–2018). Estimates from the cross-sectional and panel data fixed-effects linear probability models for the current smokers or fixed-effects models for the number of cigarettes smoked are reported. The relative income measure is calculated using the baseline definition of reference groups. Regressions include the full set of control variables: absolute and relative income (in logs), employment status (=1), working hours (in logs), marital status (married/cohabiting, single, divorced, or widowed), number of children ages 0–1, 2–4, 5–7, 8–10, 10–12, 13–15, and 16–18, household size, years of education, living in West Germany (=1), health status (5 categories), disability status, capital income or non-labour income (rent, dividend, and interest income), as well as year dummies and dummies for 16 federal states (see also Table 1). P-values are obtained from the t-test comparing two parameter estimates in (a) and (b). The magnitudes are calculated per day and multiplied by 30 days. Standard errors, clustered by reference groups, are presented in parentheses.

*, **, and *** indicate significance at 10%, 5%, and 1% levels, respectively.

Column A(II) presents the results from the asymmetrical model where we allow the relative income for the poorer (upward comparators) and the richer (downward comparators) simultaneously. In line with the previous findings, the cross-sectional specification suggests that there is a positive and highly significant association between the mean income in the reference groups and probability of smoking. The estimated coefficients of relative income for poorer and richer individuals are both positive and statistically significant with a similar magnitude. The estimated coefficients are statistically indifferent for poorer and richer individuals (p-value = 0.192). Yet, the fixed-effects specification suggests that there is basically no relationship between the absolute income or relative income and the prob- ability of smoking either in the whole sample or among the poorer or richer individuals.

#### Number of cigarettes smoked

We then investigate the relative income on the number of cigarettes smoked in the whole sample (Table 2, Columns B(I) and B(II)) and the number of cigarettes smoked among people who smoked at least once during the period 2002–2018 (Table 2, Columns C(I) and C(II)). The cross-sectional model suggests a positive and statistically precise relationship, whereas the relative income is positive but statistically insignificant with the fixed-effects model. The estimated coefficient from the cross-sectional model is about 10 times larger than that of the fixed-effects model (2.777 vs. 0.242). The interaction model specification suggests that there is no heterogeneity in the relative income estimates for poorer and richer individuals on the number of cigarettes smoked (p-value = 0.607). Yet, we also note that the relative income coefficient on the number of cigarettes smoked is only marginally significant among the poorer (upward comparators) for the whole sample. However, the relationship is again about 10 times lower (2.894 vs. 0.272) than what is estimated by the cross-sectional specification.

Finally, we investigate whether relative income relates to the number of cigarettes smoked among people who smoked at least once (Column C(I) and C(II)). As these individuals smoked at some point in their life, this sample covers the full distribution of unobserved heterogeneity among the smokers or people who are inclined to smoke. The results are highly similar to the preceding, with one important difference: the estimated association is positive and statistically significant even after allowing for the fixed effects. The estimated coefficient obtained from the fixed-effects model is about one-third of the cross-sectional estimates (3.570 vs. 1.167). This result is consistent with the idea that the income of others is associated with higher cigarette consumption among people who are somehow addicted or inclined to smoke. Another important finding is that the relative income parameter is statistically precise only among the poorer individuals who are relatively deprived (upward comparators). The fixed-effects model reveals that the difference between the estimated coefficients of the poorer and richer individuals is statistically significant at the conventional levels (p-value = 0.01), while the estimated coefficients are about the same as suggested by the cross-sectional specification. The results of the fixed-effects model are also consistent with the conjecture that individuals who are less advantaged than those in their comparison group might experience higher stress or anxiety due to an appreciation in the income of the comparable other individuals, which leads to a higher number of cigarettes smoked than among the advantaged individuals who make downward comparisons.

#### Magnitudes

To gauge the magnitude of the relationship between relative income and cigarette smoking behaviour, we calculate the increase in the probability of smoking and the number of cigarettes smoked per month related to a 10% increase in the income of comparable other individuals. The cross-sectional specification suggests a 5.1% increase (relative to the baseline smoking probability of 28.2%) in the probability of smoking for a 10% increase in the income of others. Yet, the magnitude of the relationship is negative and very close to zero with the fixed effects (Column A(I), Table 2). The number of cigarettes smoked per month (we first determine the daily change and multiply it by 30) is also highly overestimated, with the cross-sectional model suggesting about 8.33 more cigarettes/month, while the fixed-effects model suggests an increase as low as 0.32 cigarettes/month (Column B(I)). This result suggests that there is practically no effect of relative income on the number of cigarettes smoked when we consider overall population. However, focusing only on people who smoked at least once—who are a select group of individuals with respect to unobserved characteristics—the fixed-effects specification suggests that a 10% increase in the income of others relates to 3.5 more cigarettes/month (Column C(I)). This result implies that failing to allow for the fixed effects generates an estimate of the effect of relative income on smoking intensity that is about three times larger (11.7 cigarettes/month suggested by the cross-sectional specification).

### Robustness

#### Treatment of unobserved heterogeneity and estimators

Our baseline model specification is the panel data fixed-effects linear probability model, which is preferred because it accounts for the unobserved heterogeneity that is correlated with the observed characteristics. This specification choice is also supported by the Hausman test against the random-effects model specification (with p-values very close to zero), which imposes no correlation between the observed and unobserved determinants of cigarette smoking. Focusing only on the relative income coefficient, in this section, we present results from alternative estimators using different functional forms of the unobserved heterogeneity and model specification. The baseline model ignores the binary nature of the dependent variable (in Column A of Table 2) and the count nature of the number of cigarettes smoked (in Columns B and C of Table 2). In Row I of Table 3 (Column A), we present estimates from the *fixed-effects logit* model, which is a non-linear fixed-effects specification with no incidental parameters problem. The sample size used with this specification is smaller, at 32,183 observations, since this specification eliminates all individuals with no within-individual variation in smoking status. Thus, the fixed-effects logit model identifies the estimates on the probability of smoking initiation or relapse at some time point in the sampling period. The average marginal effects suggest that the relative income coefficient is positive and significant on the smoking initiation/relapse probability after allowing for the individual fixed-effects within a non-linear specification. The estimated association for a 10% increase in income is only a 1.25% increase in the mean baseline smoking probability (51.2%) of people in the final estimation sample.

**Table 3:** Robustness.

Dependent Variable:		A	B	C
		Probability of Smoking (Whole Sample)	#Cigarettes (Whole Sample)	#Cigarettes (Smoked at least once during 2002-2018)
Estimators and Treatment of Unobserved Individual Effects (with baseline reference groups)				
I	Logit fixed-effects (average marginal effects)	0.069 ***		
		(0.018)		
II	Poisson fixed-effects (exponential link function)		0.081 **	
			(0.041)	
III	Linear random effects model	0.057 ***	1.175 ***	2.329 ***
		(0.014)	(0.258)	(0.426)
IV	Linear correlated random effects with life satisfaction, risk preferences, Big–5, and locus of control	0.037 ***	0.823 ***	1.482 ***
		(0.011)	(0.208)	(0.417)
Measures of relative income position: ranks, lags, incomes, and inequality in the reference groups (with baseline reference groups)				
V	Ranks in the reference group	-0.014 **	-0.130	-0.476 *
		(0.006)	(0.114)	(0.271)
VI	Median levels as comparison points	0.002	0.186	1.165 ***
		(0.007)	(0.149)	(0.357)
VII	First lagged relative income measure (t-1)	0.001	0.182	0.992 ***
		(0.007)	(0.148)	(0.357)
VIII	Equivalised income (OECD scale)	-0.012	0.095	1.020 **
		(0.011)	(0.201)	(0.506)
IX	Windfall income (inheritance and lottery winnings)	0.001	0.221 *	0.892 ***
		(0.006)	(0.119)	(0.334)
X	Income inequality in the reference groups (baseline)	0.002	0.271 *	1.235 ***
		(0.007)	(0.150)	(0.363)
Reference group definitions				
XI	Reference groups 1	0.001	0.253	1.527 ***
	age, West Germany, gender	(0.007)	(0.162)	(0.399)
XII	Reference groups 2	0.012	0.464	1.229 ***
	age, West Germany, gender, married	(0.007)	(0.347)	(0.361)
XIII	Reference groups 3	-0.004	0.167	0.949 ***
	age, gender, federal states (16 regions), education	(0.006)	(0.105)	(0.256)

Authors’ own calculations from the SOEP (2002–2018). Each cell shows the estimates from a different regression. The relative income measure is calculated using the baseline definition of reference groups unless stated differently. Regressions include the full set of control variables. See Tables 1 and 2. Standard errors, clustered by reference groups, are presented in parentheses.

*, **, and *** indicate significance at 10%, 5%, and 1% levels respectively.

The count nature of the number of cigarettes smoked suggests a Poisson regression model (with exponential link function). The *fixed-effects Poisson* regression model eliminates all individuals who never smoked during the sampling period. Thus, the sample size is the same as the results given in Row I. Results given in Column B of Table 3 (Row II) suggest that relative income is statistically significant on the number of cigarettes smoked after allowing for the count nature of data and individual fixed effects simultaneously. This specification uses exactly the same sample where we eliminate those who never smoked (Column C, Table 3), and thus the results are omitted. The magnitude of the association is then calculated using the exponential link function which corresponds to 3.25 more cigarettes for a 10% increase in relative income. The result is highly comparable to the linear fixed-effects model in the baseline.

We then experiment with the auxiliary distribution of unobserved individual effects using linear random-effects panel data model specifications. To this end, we first start with the random-effects specification that simply imposes zero correlation between the observed and unobserved individual characteristics. As mentioned above, this model specification is not supported by the Hausman test against the baseline fixed-effects model specification. We report the results from the random-effects model in Row III of Table 3. We find that the estimated parameters are very large in magnitude and similar to those from the cross-sectional model specification. Then, we estimate correlated random-effects model specification where we condition the auxiliary distribution of the unobserved individual effects with several proxies and within means of time-variant characteristics ([34]). The proxies include the Big–5 personality characteristics ([35–37]), locus of control ([38]), life satisfaction, and subjective risk preferences ([39]). The time-variant characteristics include the health status, age, working hours, and non-labour income. The distribution of the unobserved individual effect involves the *within* means of these characteristics.

The results in Row IV suggest that the coefficient of relative income on the probability of smoking is still positive and statistically significant. We note that, the magnitude of the estimates are smaller compared to linear random-effects model specification (Row III). The estimated expected increase in the probability of smoking (among all people in the population, Column A) is about 1.68% for a 10% increase in the income of others (baseline smoking probability is 28.2%). In Columns B and C, Row IV, we estimate the effect of relative income on the number of cigarettes smoked. The correlated random-effects model with a rich unobserved heterogeneity distribution suggests that the effect of relative income on the number of cigarettes smoked is statistically significant. The magnitude of the relationship is about 2.1 cigarettes/month for the whole sample (Column B) and 5.1 cigarettes/month for the people who smoked at some point (Column C) for a 10% increase in the relative income of comparable others.

#### Measures of income, relative concerns, inequality, and reference groups

In Row V of Table 3, we present results from a model using the income *ranks* of individuals within their reference groups. To calculate the ranks, we first determine the reference groups and rank the incomes within each reference group from smallest to highest. Then we identify the income rank of each individual and normalise it to be between 0 and 1. The coefficient of income rank is expected to be negative on the probability of smoking, and the number of cigarettes smoked since a better income rank in the reference group implies a higher income status. Indeed, the effect of income rank is negative and statistically significant on the probability of smoking (Row V, Column A) and also on the number of cigarettes smoked among people who have smoked at least one cigarette (Row V, Column C). In the next specification, we change the ‘reference income’ point from mean to *median*. The median is robust especially for reference groups with smaller sample sizes. Yet, the results are highly comparable with those of the baseline (Row VI).

To check the association with respect to previous year absolute and relative income, we allow our regressions for the *first lags*. We note that income is observed annually while cigarette smoking behaviour is observed biennially. First, using lagged absolute and relative income measures does not lead to loss of observation due to lags. Second, lagged income measures might capture the potential bias due to unobserved changes in income between biennial waves. The results in Row VII suggest that the relative income is statistically significant only among the people who smoked at least one cigarette during the sampling period, which is highly consistent with the baseline results. The magnitude of the relationship is also consistent, with about 2.7 cigarettes/month for a 10% increase in the previous year’s relative income. Row VIII checks whether using *income per capita* (using OECD scale) changes the main findings. Per capita income is calculated by dividing the household income by the number of members in the household using the OECD weights as follows: $Per\;capita\;income=\frac{Household\;income}{\left(1+0.7(\#adults)+0.5(\#children\right)}$. Again, the results are highly consistent with those of the baseline.

One important bias can be generated if the absolute and relative income is endogenous in the smoking behaviour Eq (1) (e.g., reverse causality between smoking and income). Even though the fixed-effects model specification teases out potential time-invariant omitted variables correlating with the absolute and relative income, we further check this point by using the *windfall income*. This income source is expected to be more exogenous as it is obtained from unexpected gains such as lottery winnings or income inheritance. A sudden rise in the income of others and the resulting consumption might be perceived by the individuals, triggering status-related concerns. The results in Row IX suggest that the effect of relative windfall income (conditional on absolute household income and absolute windfall income) on the number of cigarettes smoked is positive and statistically significant among the whole population and also among the people who smoked at least one cigarette in the past (Row IX, Columns B and C). We check for the role of *income inequality* in the reference groups, which can independently influence the psychological outcomes of individuals. To this end, we calculate the reference groups’ specific Gini coefficients and allow in our regressions. Row X suggests that allowing our regressions for the income inequality in the reference group renders the relative income coefficient partially statistically significant for the whole sample. Overall, the estimated coefficients and statistical significance levels are highly similar to those of the baseline.

Finally, we experiment with several alternative definitions of reference groups. The baseline reference group definition (individuals living in the regions of East and West Germany, with similar age groups, gender, and education levels for each year) is modified with respect to characteristics used in the definition. In Row XI, we exclude education and use only age groups, gender, and West Germany every year. The coefficients are significant only among people who smoked at least once (Column C), similar to the results obtained with the baseline reference group definition. The parameter estimate is somehow larger with 4.2 cigarettes/month for a 10% increase in the relative income. Adding marital status into the specification generates estimates that are highly consistent estimates with those of the baseline (Row XII). In the last row, we investigate the role of the size of regional units used in the reference groups. Indeed, the regional units might play an important role if there is significant regional heterogeneity in the attitudes towards smoking or in the sources of income and income distributions. In Row XIII, we replace 16 federal states in our baseline reference group definition. The results are somehow smaller than those of the baseline, yet the pattern and overall implications are the same.

## Conclusion

This paper investigates how status concerns relate to cigarette smoking behaviour using long panel data with which we can allow for individual fixed effects correlated with reference group formation, absolute and relative income, and cigarette smoking behaviour. The results suggest that unobserved individual characteristics play an important role in the relationship between status concerns and smoking. Failure to consider them leads to a highly overestimated relationship and causal interpretations that are highly limited. The fixed-effects model that accounts for time-invariant heterogeneity, which is expected to differ between smokers and non-smokers, suggests that there is basically no relationship (or a very weak relationship, as suggested by some specifications) between the relative concerns and smoking behaviour among the whole population. The relationship suggested in the literature is also not supported for relatively deprived individuals (i.e., poorer individuals who make upward comparisons).

For a subset of individuals who have smoked at least once in their life (at least during the period 2002–2018) and are already addicted to nicotine or are inclined to smoke, accounting for the unobserved heterogeneity suggests a significant effect of relative income on the probability of smoking (initiation or relapse) or the number of cigarettes smoked. Among this group of individuals, a 10% increase in the income of others relates to only 3.5 more cigarettes/month. Our findings imply that the results suggested by the cross-sectional model specification are biased, as it highly overestimates the association between relative income and smoking behaviour and incorrectly identifies which groups of individuals are actually affected. Our study contributes to this small literature by econometrically investigating the relationship in more detail and providing insights as to for whom and in what extent relative income influences cigarette smoking behavior, a major public health concern. One important implication is that the cross-sectional results might lead to incorrect public policy conclusions, and studies aiming to conduct health-related economic calculations should take these findings into account.
